# Case Report: Successful Management of a Ruptured Abdominal Aortic Aneurysm With Concurrent Acute Myocardial Infarction in an 84‐Year‐Old Male

**DOI:** 10.1002/ccr3.73127

**Published:** 2026-08-19

**Authors:** Ali Ghatfan, Maisam Taherian, AliAsghar Zafar Asoodeh, AliReza Farzaei

**Affiliations:** ^1^ School of Medicine Aja University of Medical Sciences Tehran Iran; ^2^ Interventional Cardiologist, Madaen Hospital Tehran Iran; ^3^ Anesthesiologist, Madaen Hospital Tehran Iran; ^4^ School of Medicine Shahid Beheshti University of Medical Sciences Tehran Iran

**Keywords:** coronary artery disease, EVAR, multidisciplinary management, open surgical repair, ruptured AAA

## Abstract

Rupture of an abdominal aortic aneurysm (AAA) is a catastrophic event with mortality rates historically approaching 80%, often compounded by significant coronary artery disease. We present the challenging case of an 84‐year‐old man who presented with a contained AAA rupture and a synchronous acute myocardial infarction. Given his hemodynamically stable but precarious state, our team prioritized immediate percutaneous coronary intervention with drug‐eluting stents, which mandated dual antiplatelet therapy (DAPT). An ensuing attempt at endovascular repair (EVAR) failed because of extensive iliac calcification and a femoral artery injury that occurred during access. Consequently, open surgical repair was undertaken on hospital Day 5 despite ongoing DAPT—a high‐risk maneuver that proceeded without major bleeding complications. This exceptional outcome underscores that, in select patients with a contained rupture, a strategy of prioritized coronary revascularization followed by open repair while on DAPT can be successful, highlighting the vital importance of adaptable, multidisciplinary decision‐making. At 2.5 years, the patient remains alive and functionally independent, confirming the durability of this approach.

## Key Clinical Message

There is no algorithm for a contained ruptured AAA with concurrent myocardial infarction. This case does not propose a solution but illustrates the extreme trade‐offs involved, questioning whether traditional surgical contraindications must be re‐evaluated when managing such complex, life‐threatening conditions.

## Graphical abstract

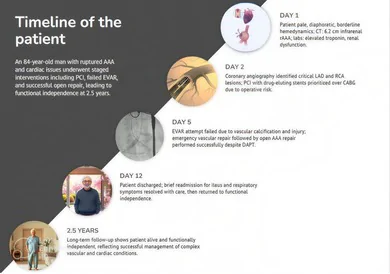

## 1 Introduction

Ruptured abdominal aortic aneurysm (rAAA) is a lethal condition requiring immediate intervention, with mortality rates still exceeding 50% despite advances in care [1, 2]. This emergency becomes a profound clinical dilemma when it coincides with acute coronary syndrome (ACS), a common comorbidity in AAA patients [3].

In such scenarios, the standard imperative for immediate aortic repair directly conflicts with the need to address life‐threatening cardiac ischemia. The dilemma is intensified if percutaneous coronary intervention (PCI) is performed, as the mandatory dual antiplatelet therapy (DAPT) creates a significant barrier to subsequent major surgery [4, 5]. No established guideline exists for managing this high‐stakes conflict.

We present the exceptional case of an octogenarian with a contained rAAA and synchronous ST‐elevation myocardial infarction. This report describes the successful, staged management involving prioritized PCI with DAPT followed by open surgical repair, demonstrating that a multidisciplinary, adaptive strategy can achieve a positive outcome in this extreme‐risk situation.

## 2 Case History

An 84‐year‐old man was urgently transferred to our tertiary care center after a bedside ultrasound at a peripheral hospital raised suspicion of a ruptured abdominal aortic aneurysm (rAAA). On arrival, he appeared pale and diaphoretic, reporting both abdominal and retrosternal chest pain. Despite these symptoms, his hemodynamics were relatively stable, with a systolic blood pressure hovering around 100 mmHg. He was admitted to the intensive care unit for close monitoring, fluid resuscitation, and preparation for potential surgical intervention.

**FIGURE 1:**
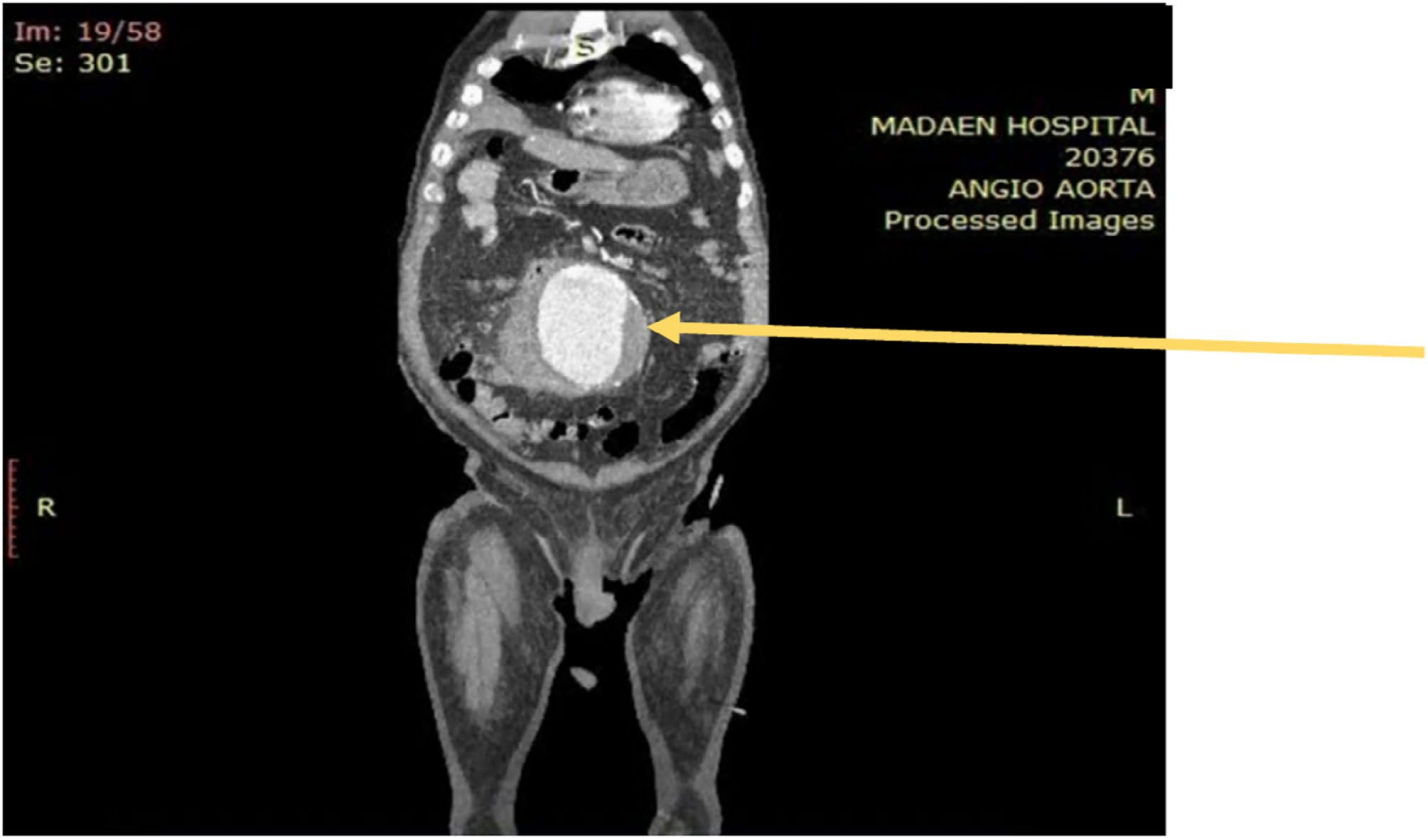
Coronal CT scan, ruptured and sealed‐off abdominal aortic aneurysm; shown by arrow.

Initial investigations revealed a contained rupture of a 6.2 cm infrarenal abdominal aortic aneurysm with a retroperitoneal hematoma on contrast‐enhanced CT (Figure 1). Transthoracic echocardiography showed mild left ventricular dysfunction (LVEF ~45%) and regional wall motion abnormalities. Laboratory studies were notable for elevated troponin I (2 ng/mL), anemia (Hb 11.2 g/dL), leukocytosis (WBC 17,800/mm^3^), and impaired renal function (creatinine 2.6 mg/dL). A detailed day‐by‐day table of the patient's laboratory data is provided (Table 1). These findings raised concern for a concurrent acute coronary syndrome, prompting urgent coronary angiography.

**TABLE 1:** Serial laboratory values during hospitalization.

	23 Feb 2023	24 Feb	25 Feb	26 Feb	27 Feb	28 Feb	29 Feb	6 Mar	7 Mar
WBC (Neutrophil)	17,800 (82%)	21,400 (82%)	13,400 (80%)	12,500 (88%)	11,000 (81%)	11,100 (82%)	10,900 (86%)	14,900 (84%)	12,800 (84%)
Hb	11.2	9.8	9	9.3	9.3	9.1	10	10	9.6
Plt	145,000	158,000	140,000	333,000	146,000	142,000	104,000	108,000	101,000
Creatinin	2.65	2.6	2.1	1.52	1.46	1.43	1.62	2.38	2.24
Troponin I	2 ng/mL								
Albumin	4.1 g/dL								
D‐Dimer	7877								
CRP	180 mg/L								
Covid 19 PCR			Negative						
Sputum culture					Negative				
AST	18						35		
ALT	15						18		
Alkaline phosphatase	227						126		
Blood culture	Negative	Negative	Negative						

*Note:* Detailed proper laboratory data is provided here. Coronary angiography is performed on day 23 February. Coronary intervention is performed on day 25 February and unsuccessful EVAR was done on day 28 February. March 7 is discharge day.

## 3 Investigations and Treatment

**FIGURE 2:**
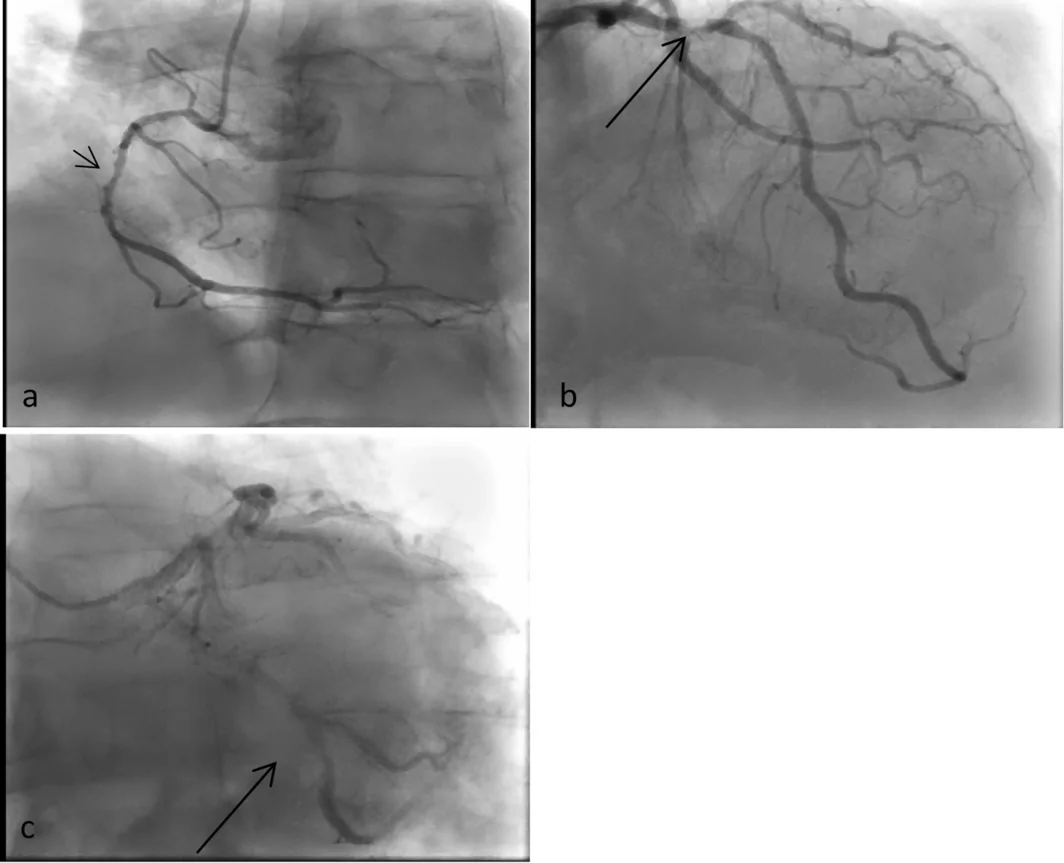
Severe stenosis in middle of RCA (a) and LAD (b) arteries. RCA lesion seems to be thrombotic and fresh. The coronary system is co‐dominant (c).

**FIGURE 3:**
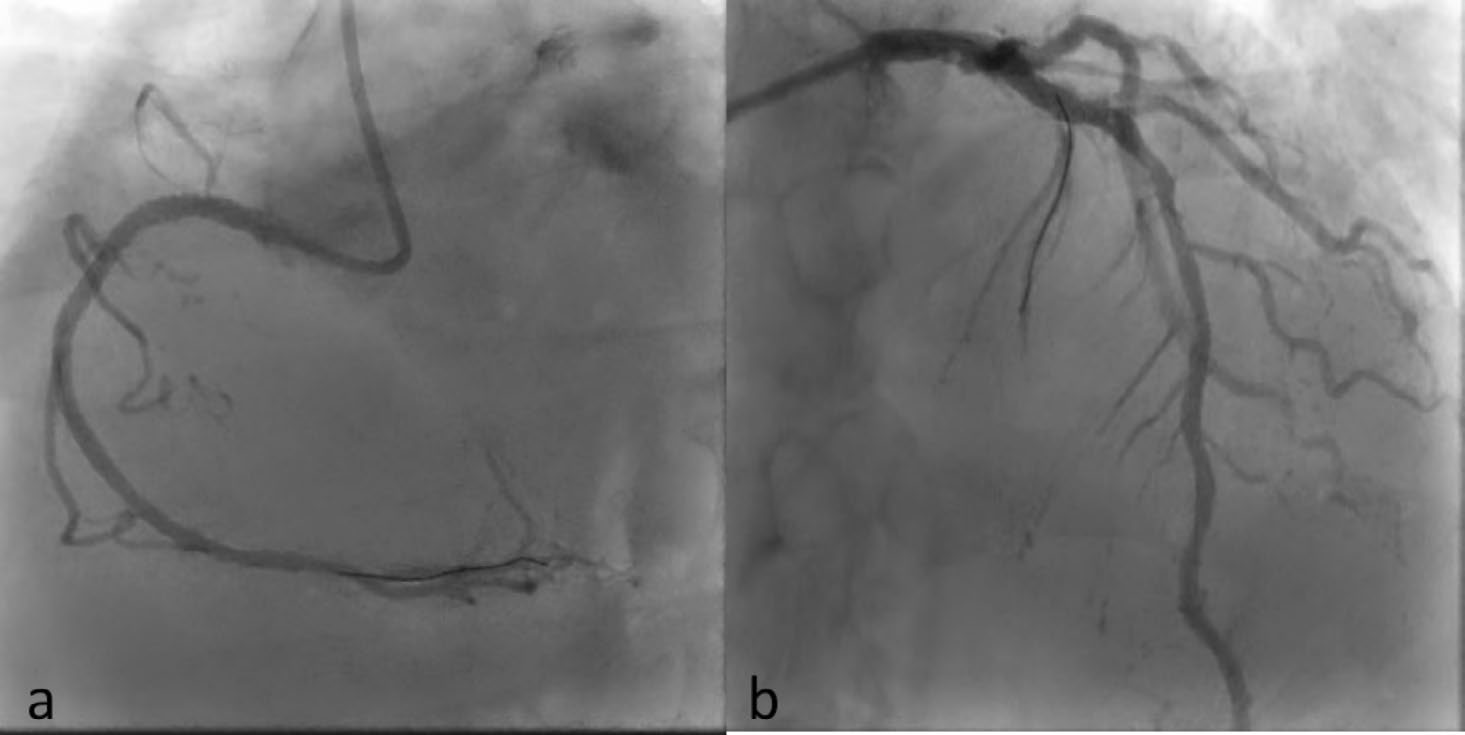
RCA (a) and LAD (b) after percutaneous coronary intervention. Their stenosis has been resolved because of the intervention.

On hospital Day 2, angiography revealed significant mid‐left anterior descending artery stenosis and thrombotic critical stenosis of the mid‐right coronary artery (Figure 2). Given the patient's stable hemodynamics and the contained nature of the aneurysm, the multidisciplinary team prioritized coronary revascularization to reduce the immediate risk of perioperative cardiac death. An urgent cardiology consultation was obtained. The heart team debated the risks of PCI versus CABG; because of the technical complexity and surgical risk, the option of combined coronary artery bypass grafting (CABG) and open AAA repair was deferred, and PCI was chosen. Percutaneous coronary intervention was successfully performed with a drug‐eluting stent (Figure 3), and dual antiplatelet therapy (DAPT) was initiated.

**FIGURE 4:**
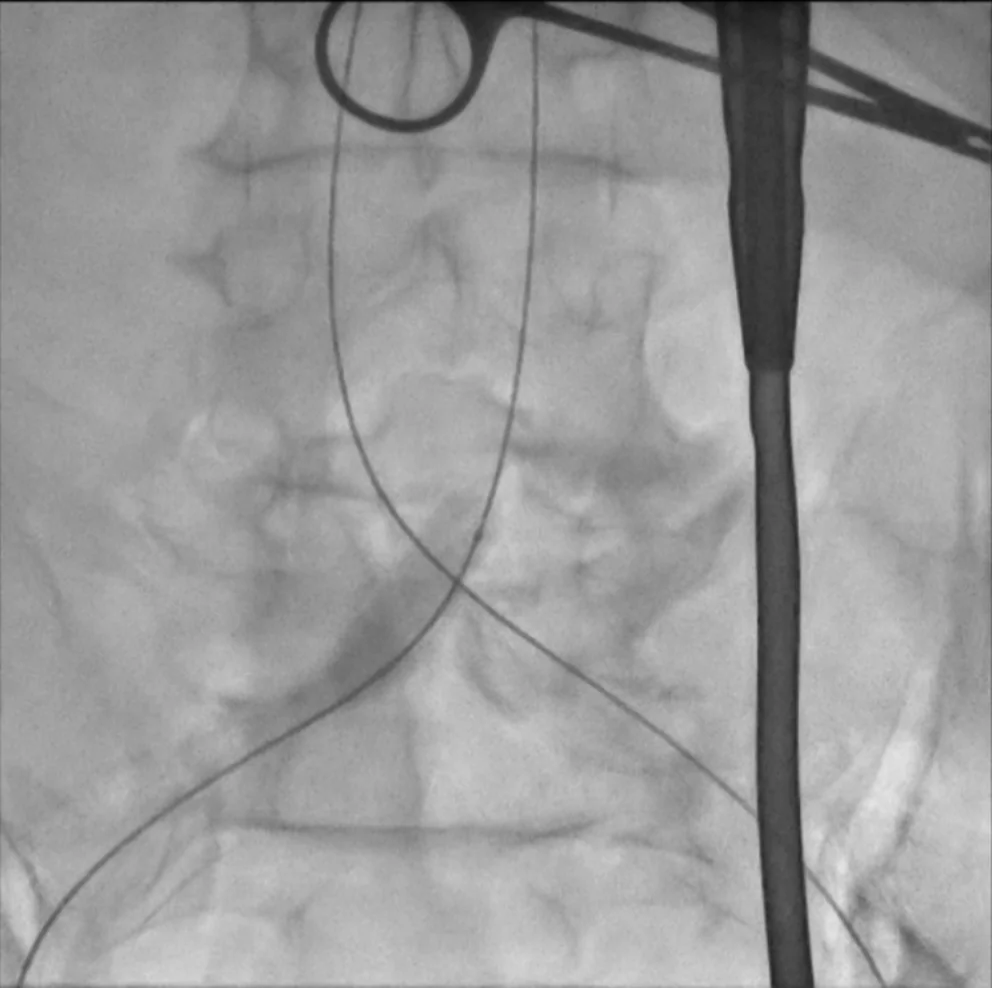
A scene from failed EVAR procedure.

Following PCI, the team planned for endovascular aneurysm repair (EVAR) on Day 5. CT angiography revealed severe bilateral iliac artery stenosis and extensive calcification, necessitating stent‐assisted access. Preprocedural planning included detailed iliac measurements: the right external iliac artery had a minimal luminal diameter of 7 mm, severe circumferential calcification, and mild tortuosity, indicating a challenging access situation. Because the patient was on mandatory DAPT, EVAR was chosen over open surgery to limit bleeding risk, accepting the access difficulties and planning for predilation, stent‐assisted access, and a surgical cut‐down. In hindsight, alternative strategies—such as proceeding directly to open repair or creating an iliac conduit—should have been given greater consideration. A Cook Zenith Flex iliac limb stent graft system with a 13 mm distal diameter was selected. Low‐profile systems were available but were deemed unsuitable: the Ovation device requires adequate infrarenal neck length and compliance, while the Incraft system's maximum distal iliac limb diameter could not accommodate the 13 mm common iliac landing zone; both also employ longer delivery systems, which were judged higher risk in the setting of a contained rupture. Even a sheath would have exceeded the 7 mm lumen, and the circumferential calcification would have prevented safe advancement. Access was obtained via a right common femoral surgical cut‐down. Predilation was performed with balloons, followed by placement of a balloon‐expandable covered stent across the stenotic segment. Despite these maneuvers, the sheath could not be advanced beyond the mid‐external iliac artery; repeated attempts caused femoral artery transection. No iliac conduit was attempted because no healthy segment was available for anastomosis, and the EVAR procedure was aborted (Figure 4). The mechanism of failure was clear: the 7 mm lumen was well below the sheath's outer diameter, and the circumferential calcification created a rigid, non‐compliant conduit resistant to dilation. Calcification extended into the common femoral artery at the puncture site—a finding initially accepted but underappreciated for the risk of transection under the forces required for sheath advancement. Emergency vascular repair of the femoral injury was performed, and the patient was stabilized.

Later that same day, open surgical repair of the aneurysm was undertaken despite the ongoing DAPT. The decision to proceed was based on the patient's overall stability and the inability to achieve endovascular control. Remarkably, the surgery was completed without major bleeding or perioperative complications.

**FIGURE 5:**
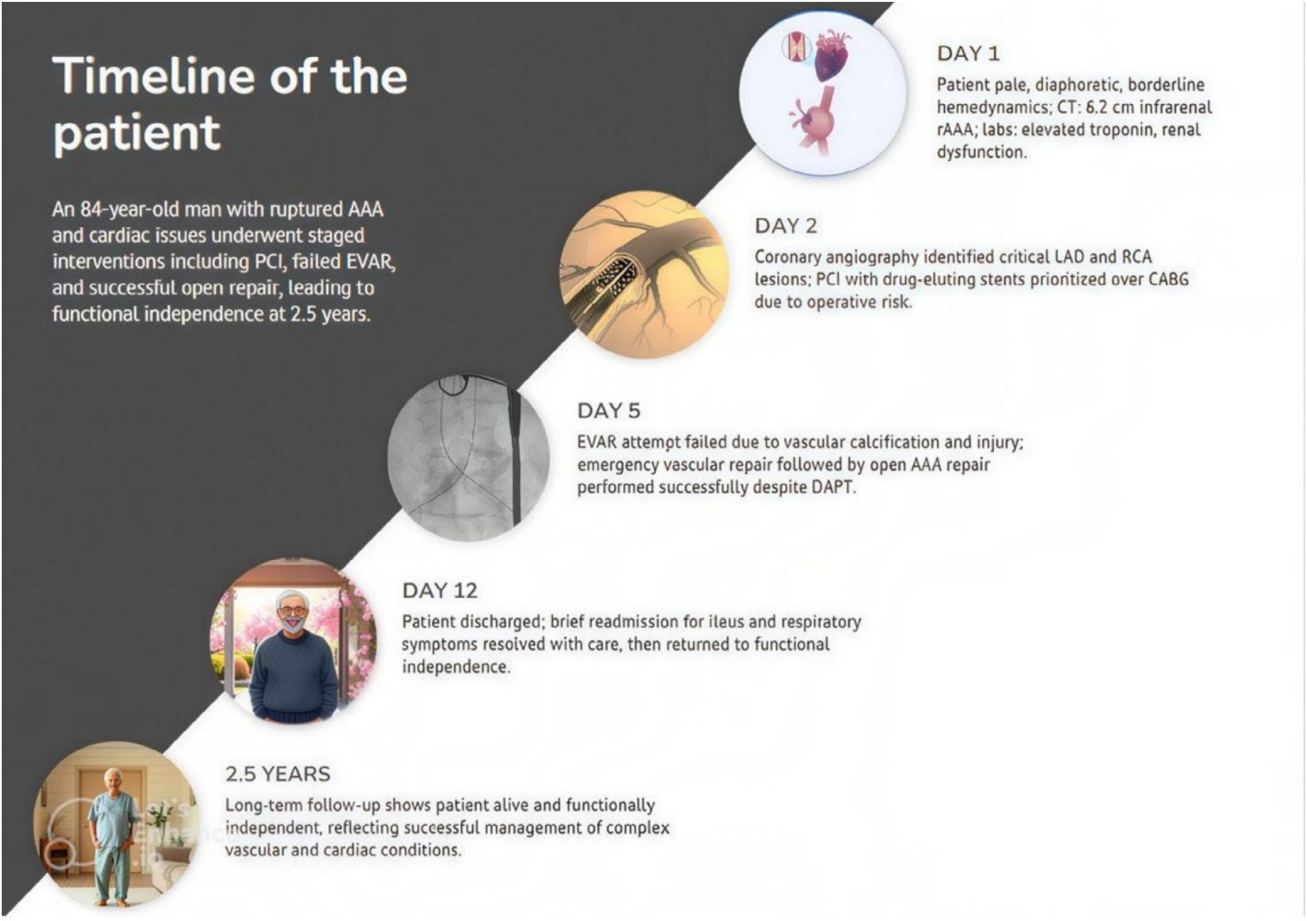
Patient's course from presentation through hospitalization and to the discharge day important events are mentioned.

The patient recovered uneventfully and was discharged on postoperative Day 7. He experienced a brief readmission at an outside facility for ileus and respiratory symptoms, which resolved with supportive care. At 2.5‐year follow‐up, he remains alive and functionally independent, underscoring the success of a carefully staged, multidisciplinary approach in managing this complex vascular emergency. A timeline summarizing the patient's course is provided (Figure 5).

## 4 Discussion

This report describes an 84‐year‐old male with rAAA and concurrent critical coronary artery disease (CAD), a high‐risk scenario that demanded multidisciplinary coordination. The patient's hemodynamic instability, elevated troponins, and echocardiographic wall motion abnormalities indicated acute coronary syndrome (ACS), complicating any immediate aneurysm repair.

Proceeding with open aortic repair under mandatory dual antiplatelet therapy (DAPT) represented our most significant deviation from standard practice. Conventional wisdom mandates stopping P2Y12 inhibitors before major surgery to mitigate bleeding risk [4, 5]. However, in this life‐threatening scenario, the risk of fatal stent thrombosis from discontinuing DAPT acutely after STEMI—which can exceed 40%—far outweighed the manageable risk of surgical bleeding [6]. Emerging evidence from complex vascular cases supports this calculus, indicating that while bleeding complications increase, mortality is more closely linked to cardiac events than to hemorrhage when meticulous surgical technique is employed [7]. Our successful outcome demonstrates that with a multidisciplinary strategy, accepting the DAPT challenge can be a necessary and viable pathway to survival.

Our decision to delay definitive AAA repair for 5 days to allow for prior coronary revascularization challenges the principle of immediate intervention for rupture. This approach was predicated on the critical distinction of a contained rupture, which can provide a temporary but precarious window of stability. While delay is generally associated with increased mortality, recent analyzes suggest that selected patients with contained hematomas and hemodynamic stability may be managed with a brief delay, potentially allowing for optimization of critical comorbidities [8].

Despite planning for EVAR, severe iliac artery stenosis and circumferential calcification precluded stent‐graft deployment, leading to femoral artery transection during access. This complication underscores the anatomical limitations of EVAR in calcified vasculature [9]. Even with a stent‐assisted approach to facilitate access, the stent graft could not be advanced because of the extreme anatomic difficulty. Papadoulas et al. described a bailout technique for EVAR delivery systems obstructed at the flow‐divider level, using a bidirectional force maneuver with a contralateral molding balloon to disengage the device while minimizing graft migration [10]. Although such troubleshooting strategies may be effective when the endograft is partially deployed, they are not applicable when the device cannot be advanced into the vasculature, as occurred in our patient.

The patient's 2.5‐year survival with good functional status is remarkable given the > 50% 1‐year mortality typically seen in octogenarians with rAAA [8].

Our management strategy shared similarities with hybrid approaches combining EVAR and open common femoral artery repair, as described by Papadoulas et al. for concomitant aortoiliac and femoral aneurysms [11]. In our case, PCI was prioritized because of the immediate risk of perioperative cardiac mortality, despite the subsequent challenge of performing major vascular surgery under ongoing DAPT. Although EVAR was initially planned to minimize bleeding risk associated with open surgery during DAPT, severe iliac calcification and difficult vascular access led to procedural failure and femoral artery transection. This experience highlights both the importance of secure vascular access in hybrid endovascular procedures and the need for timely conversion to open repair when endovascular intervention becomes unsafe or technically unfeasible.

Our failed EVAR attempt also highlights the long‐term limitations of endovascular repair in hostile iliac anatomy. Papadoulas et al. identified type I endoleak as the leading cause of late post‐EVAR rupture, particularly in patients with inadequate sealing zones and severe iliac disease [12]. In our patient, marked iliac calcification and stenosis not only prevented device delivery but would also have increased the risk of distal seal failure, graft thrombosis, and late aneurysm rupture. These concerns are especially relevant in the setting of ongoing DAPT, which may complicate future reinterventions. Consequently, although the initial EVAR attempt failed, conversion to definitive open repair likely provided a more durable long‐term solution in this anatomically unfavorable and high‐risk patient.

Rare vascular pathologies, including superficial venous aneurysms, also present significant diagnostic and therapeutic challenges. As reviewed by Skandali et al., these uncommon lesions are frequently misdiagnosed and lack standardized management guidelines, despite risks of thrombosis or rupture [13]. Like our case, management requires an individualized and multidisciplinary approach that balances procedural feasibility against the immediate risks of vascular complications.

## 5 Conclusion

This case affirms that multidisciplinary, adaptive decision‐making can achieve success in extreme‐risk vascular emergencies, justifying prioritized revascularization and open repair despite antiplatelet therapy. This case also emphasizes that contained rupture may allow a narrow window for preoperative cardiac optimization, and that hybrid bailout techniques for difficult iliac access should be part of the surgical armamentarium, even when primary EVAR is aborted. Future efforts must focus on developing better strategies for hostile anatomy.

## Author Contributions

**Ali Ghatfan:** conceptualization, methodology, project administration, supervision, visualization. **AliAsghar Zafar Asoodeh:** conceptualization, supervision. **AliReza Farzaei:** conceptualization, writing – original draft, writing – review and editing. **Maisam Taherian:** project administration.

## Funding

The authors have nothing to report.

## Ethics Statement

Informed consent was obtained from the patient for publication of this case report and any accompanying images; the Helsinki 1964 Declaration and its later amendments were observed.

## Consent

The patient gave written informed consent to publish this report in accordance with the journal's patient consent policy.

## Conflicts of Interest

The authors declare no conflicts of interest.

## Data Availability

The data that support the findings of this study are available on request from the corresponding author. The data are not publicly available due to privacy or ethical restrictions.Data Availability Statement: Data sharing is not applicable to this article as no datasets were generated or analyzed during the current study. All relevant clinical information is contained within the manuscript.
